# Selective Ablation of Dehydrodolichyl Diphosphate Synthase in Murine Retinal Pigment Epithelium (RPE) Causes RPE Atrophy and Retinal Degeneration

**DOI:** 10.3390/cells9030771

**Published:** 2020-03-21

**Authors:** Marci L. DeRamus, Stephanie J. Davis, Sriganesh Ramachandra Rao, Cyril Nyankerh, Delores Stacks, Timothy W. Kraft, Steven J. Fliesler, Steven J. Pittler

**Affiliations:** 1 Department of Optometry and Vision Science, Vision Science Research Center, University of Alabama at Birmingham, Birmingham, AL 35294, USA; marcismith1952@gmail.com (M.L.D.); stephanie.davis@bison.howard.edu (S.J.D.); cyrilnya@uab.edu (C.N.); delorez@uab.edu (D.S.); twkraft@uab.edu (T.W.K.); 2 Departments of Ophthalmology and Biochemistry, State University of New York-University at Buffalo, Buffalo, NY 14209, USA; and Research Service, VA Western NY Healthcare System, Buffalo, NY 14215, USA; sramacha@buffalo.edu (S.R.R.); fliesler@buffalo.edu (S.J.F.)

**Keywords:** retinal degeneration, retinitis pigmentosa, retinal pigment epithelium dystrophy, RPE transmigration, Cre-Lox technology, mouse models

## Abstract

Patients with certain defects in the dehydrodolichyl diphosphate synthase (DHDDS) gene (RP59; OMIM #613861) exhibit classic symptoms of retinitis pigmentosa, as well as macular changes, suggestive of retinal pigment epithelium (RPE) involvement. The DHDDS enzyme is ubiquitously required for several pathways of protein glycosylation. We wish to understand the basis for selective ocular pathology associated with certain DHDDS mutations and the contribution of specific ocular cell types to the pathology of mutant *Dhdds*-mediated retinal degeneration. To circumvent embryonic lethality associated with *Dhdds* knockout, we generated a Cre-dependent knockout allele of murine *Dhdds* (*Dhdds^flx/flx^*). We used targeted Cre expression to study the importance of the enzyme in the RPE. Structural alterations of the RPE and retina including reduction in outer retinal thickness, cell layer disruption, and increased RPE hyper-reflectivity were apparent at one postnatal month. At three months, RPE and photoreceptor disruption was observed non-uniformly across the retina as well as RPE transmigration into the photoreceptor layer, external limiting membrane descent towards the RPE, and patchy loss of photoreceptors. Functional loss measured by electroretinography was consistent with structural loss showing scotopic a- and b-wave reductions of 83% and 77%, respectively, at three months. These results indicate that RPE dysfunction contributes to DHDDS mutation-mediated pathology and suggests a more complicated disease mechanism than simply disruption of glycosylation.

## 1. Introduction

Retinitis pigmentosa (RP) and related disorders are characterized by degeneration and loss of photoreceptors, attenuation of retinal blood vessels, pigment deposits, and a waxy pallor of the optic disc that result in impaired night vision and peripheral and central vision loss [1]. Defective protein glycosylation in the retinal pigment epithelium (RPE) has been associated with retinal degeneration elicited by photoreceptor abnormalities and impaired phagocytosis of aging photoreceptor outer segment (OS) membranes [2,3,4,5]. One such example, dehydrodolichyl diphosphate synthase (DHDDS; OMIM #608172), is an essential enzyme in the mevalonate pathway where it functions ubiquitously in isoprenoid chain elongation to form dolichols that comprise 17–20 isoprene units. The phosphorylated form of dolichol, dolichol pyrophosphate is necessary for N-glycosylation at specific residues in many membrane proteins [6]. There is no known unique function for DHDDS in the retina, or in any other ocular tissue. There are numerous N-glycosylated proteins in the retina and photoreceptors, such as rhodopsin [7] and the cGMP-gated cation channel in rods. Additionally, in the RPE, there are several glycosylated structural proteins, ion channels and transport proteins that contribute to the transepithelial potential of the polarized RPE monolayer [8,9,10].

Mutations in the gene encoding DHDDS lead to a recessive form of RP called RP59 (OMIM #613861, DHDDS, K42E), which was first identified in several families of Ashkenazi Jewish origin [11,12,13]. RP59 is considered to belong to the family of human genetic diseases known as “congenital disorders of glycosylation” (CDGs) [14]. Two other mutations in the DHDDS gene (T206A and R98W), which occur heterozygously with the K42E mutation, were also reported, but have not been studied in detail [15,16]. Patients with the K42E mutation in DHDDS exhibit all the cardinal features of RP as well as macular changes [12], suggesting possible RPE involvement. While the K42E, T206A, and R98W mutations are only associated with known RP symptoms, another mutation in the DHDDS gene has led to infant morbidity at seven months of age [17].

As a first step to assess the role of DHDDS in specific retinal cell types and to understand the molecular mechanism of RP59, we created a Cre-lox dependent line of mice that allows targeted, cell type-specific deletion of *Dhdds* in cells of interest. Using a conditional knockout was necessary to circumvent embryonic lethality associated with global knockout of *Dhdds* [18,19]. Here, we describe the generation and characterization of a *Dhdds^flx/flx^* CreRPE mouse line (i.e., RPE-specific *Dhdds* knockout) and its validation as a model of RPE atrophy and retinal degeneration. We show that RPE-specific deletion of *Dhdds* induces structural and functional deficits in the RPE and the photoreceptors, which suggests that RPE pathology may be a significant contributor to the retinal degeneration observed in patients with RP59 mutations.

## 2. Materials and Methods

### 2.1. Generation of Dhdds^flx/flx^ CreRPE Mice

A construct containing lacZ flanked by FLP-FRT and *Dhdds* exon 3 flanked by loxP sites from the Knockout Mouse Project (KOMP, UC Davis, Davis, CA, USA) was linearized and introduced into mouse ES cells (C57Bl/6J background) at the Roswell Park Cancer Institute (RPCI) Gene Targeting and Transgenic Facility (Buffalo, NY, USA) using standard technology. To confirm the correctly targeted cells, polymerase chain reaction (PCR) was performed with the primers listed below (see *PCR Genotyping*, below). The lacZ cassette was excised with FLP-FRT recombinase, and excision was confirmed by PCR. Mouse lines that carried the *Dhdds* loxP conditional knockout allele were crossed to generate homozygotes and the latter were also crossed to a mouse line (on a C57Bl/6J background) carrying a homozygous transgene expressing Cre recombinase under the control of the RPE-specific VMD2 (vitelliform macular degeneration 2) promoter [20,21]. RPE-specific expression of Cre in the VMD2 promoter-driven mouse line was confirmed by crossing those mice with a ZsGreen reporter mouse line (B6.Cg-*Gt(ROSA)26Sor^tm6(CAG-ZsGreen1)Hze^*/J, The Jackson Laboratory, Bar Harbor, ME, USA) and examining the retinas by confocal fluorescence microscopy. Genotypes of offspring were confirmed by PCR with *Dhdds-* and Cre RPE-specific primer pairs. All mice used in this study were treated following the ARVO *Statement on the Use of Animals in Ophthalmic and Vision Research* and the policies of the University of Alabama at Birmingham (UAB) Institutional Animal Care and Use Committee (IACUC). This project was approved for animal use on April 2019 by the UAB IACUC and requires updated approvals each year (protocol number IACUC-21270). All animals were maintained on a standard 12/12-h light/dark cycle, fed standard rodent chow, provided water *ad libitum*, and housed in plastic cages with standard rodent bedding.

### 2.2. PCR Genotyping

Mouse genomic DNA samples obtained from tail snips were verified by PCR using primers *Dhdds*-FWD: 5′-GTGTCATCCCCTGCTGCAGAT-3′ and *Dhdds*-REV: 5′-TGGGTGTAGTG-GCTCAGGTC-3′ for genotype identification of floxed *Dhdds* alleles designed in a region which was conserved in both wild type (WT) and floxed alleles and also in the region flanking the loxP sites. The expected PCR product sizes for the WT and floxed alleles are 393 and 517 bp, respectively, thus differentiating WT, heterozygous floxed, and homozygous floxed alleles. PCR verification of Cre transgene modification was carried out using the following forward and reverse primer sets for Cre RPE 5′-AGGTGTAGAGAAGGCACTTAGC-3′ and 5′-CTAATCGCCATCT-TCCAGCAGG-3′, respectively, yielding a 411 bp product. RPE specific expression and activity of Cre-recombinase in VMD2-RPE Cre was verified by breeding these mouse lines against an ZsGreen reporter mouse strain (B6.Cg-*Gt(ROSA)26Sor^tm6(CAG-ZsGreen1)Hze^*/J, Stock# 007906; The Jackson Laboratory, Bar Harbor, ME, USA) and monitoring ZsGreen expression in the retina. 

### 2.3. Spectral-Domain Optical Coherence Tomography

Spectral-domain optical coherence tomography (SD-OCT) (840 nm; Bioptigen, Inc./Leica, Durham, NC, USA) was used to obtain in vivo images of the retina at one, two, and three months of age. OCT images were collected with Bioptigen InVivoVue^®^ 1.4 software and Bioptigen Diver^®^ 2.0 software was used to analyze outer nuclear layer (ONL) thickness and full retinal thickness (F.R.T.) across all retinal layers at five eccentricities spanning two-thirds of the retina centered at the optic nerve head. A detailed description of this procedure has been reported previously [22]. F.R.T. was calculated from the difference of markers 1 and 10 and ONL thickness was calculated from the difference between 5 and 6.

### 2.4. Fundus Examination and Fluorescein Angiography

Fundus examination was performed with a Micron IV digital fundus microscope (Phoenix Technology Group, Pleasanton, CA, USA), using the mouse objective and hydroxypropylmethylcellulose 2.5% on the surface of the cornea. Digital images of the fundus were captured with dedicated StreamPix^®^ 6 digital software (NorPix, Inc., Montreal, Quebec, Canada) and processed using Adobe^®^ Photoshop^®^ 6 (Adobe Systems, Inc., San Jose, CA, USA).

### 2.5. Visual Function Testing

Full-field scotopic and photopic electroretinograms (ERGs) were obtained as described in detail previously using an OcuScience^®^ HMsERG instrument (OcuScience, Henderson, NV, USA) [22]. Response amplitudes and implicit times of a-waves and b-waves representing the activity of photoreceptors and bipolar cells, respectively, were quantified. In brief, mice were dark-adapted overnight, anesthetized, and then placed on a heating pad to maintain body temperature. Following pupil dilation with 2.5% phenylephrine and 1% tropicamide ophthalmic solutions, a thin silver wire electrode was placed on the cornea (interfaced with methylcellulose and covered with a specially designed contact lens) and referenced to a needle ground electrode in the cheek. The responses to Ganzfeld flash stimuli, spanning a range of five log units, were measured and recorded; following light adaptation to background illumination, cone-driven responses also were recorded.

### 2.6. Histology/Immunohistochemistry 

The methodologies used in this study have been described in detail previously [23]. Briefly, for conventional histology, eyes (n ≥ 4 per condition) were fixed by immersion in freshly prepared 4% paraformaldehyde in 0.125 M Na-phosphate buffer, pH 7.4, at 4 °C overnight, embedded in paraffin, and tissue sections (toluidine blue-stained) were viewed with an Olympus BH2 photomicroscope equipped with a Nikon digital camera. Digitized images were collected and further analyzed with ImagePro Plus^®^ software, Version 4.1 (Media Cybernetics; Rockville, MD, USA). For immunohistochemistry, frozen sections of retinal tissue (embedded in Tissue-Plus™ Optimal Cutting Temperature (O.C.T.) compound; Thermo Fisher Scientific, Waltham, MA, USA), obtained with a cryostat and collected on glass microscope slides were incubated with suitable primary antibodies, with detection by application of species-specific, fluor-conjugated secondary antibodies, counterstaining nuclei with DAPI, followed by laser confocal immunofluorescence microscopy (Leica TCS SPE scanning confocal microscope; Leica Microsystems Inc., Buffalo Grove, IL, USA), as previously reported [24].

### 2.7. Fluorescein Angiography

Fluorescein angiography was performed following intraperitoneal injection (i.p., 10 μL/gram body weight) of a 10 mg/mL solution of AK-Fluor 10% (Sigma Pharmaceuticals; Liberty, IA, USA) in PBS. Uptake of fluorescein and fundus imaging was monitored with a Micron IV Retinal Imaging Microscope (Phoenix Technology Group, Inc., Pleasanton, CA, USA).

### 2.8. Electron Microscopy

Mouse eyes were processed for plastic embedment, ultramicrotomy, and EM analysis essentially as described in detail previously [23]. Immediately after sacrifice, eyes were orientated by marking the superior hemisphere along the vertical meridian at the limbus with a hot needle, before starting the dissection. A cut was made in the superior cornea and the eyes were fixed for 2 h at 4 °C in fresh 0.1 M sodium phosphate buffer (pH 7.4), containing 2.5% (v/v) glutaraldehyde, 2.0% formaldehyde and 0.025% CaCl_2_. After a 20–30 min primary fixation, the superior cornea and lens were removed, and fixation was continued overnight. The fixed eyes were then rinsed with 0.1 M sodium cacodylate buffer (pH 7.4) containing 0.025% CaCl_2_, and then post-fixed for 1 h in 1% osmium tetroxide in 0.1 M sodium cacodylate buffer. After post-fixation, the eyes were rinsed twice in 0.1 M sodium cacodylate buffer and once in distilled water, then dehydrated in graded ethanol series followed by propylene oxide and infiltration overnight in Spurr’s resin. The eyes were then embedded in resin-filled BEEM^®^ capsules (Polysciences, Warrington, PA, USA) and allowed to polymerize in a 70 °C oven for 48 h. Tissue sections were obtained with a Reichert–Jung Ultracut E^®^ microtome using a diamond knife. Thin (60–80 nm thickness) sections were collected on copper 75/300 mesh grids and stained with 2% (v/v) uranyl acetate and Reynolds’ lead citrate. Sections were viewed with a JEOL 100CX electron microscope at an accelerating voltage of 60 keV.

### 2.9. Serial Block-Face Scanning Electron Microscopy (SBF-SEM)

Samples prepared for TEM as described above were further processed by Thermo Fisher Scientific (Waltham, MA, USA) using an Apreo VolumeScope™ serial block-face scanning electron microscope (SBF-SEM). Excess resin was removed from the tissue using a Leica ultramicrotome. The trimmed blocks were then glued to a SEM stub (Agar Scientific, AGG1092450) using a two-component silver conductive epoxy, H20E EPO-TEK (Ted Pella, Inc.; Redding, CA, USA). To minimize charging of the block by the electron beam, the bottom and sides of the block were sputter-coated with a 30 nm thick gold film layer. The samples were then imaged on the VolumeScope™ operating in low vacuum mode at 50 Pa and using a lens mounted backscattered detector. All of the data sets were imaged with an accelerating voltage of 2.2 kV and a beam current of 100 pA using 1-µs dwell time combined with two-line integration. Two regions of interest (ROIs) were acquired on the knock-out sample (KO-148). For ROI1, 738 sections were collected with the internal microtome set to a 40 nm cutting thickness (z resolution) with an area of 92.9 µm × 89.1 µm at 10 nm/pixel. For ROI2, 745 sections were collected with an area of 97.6 µm × 96.3 µm using the same imaging condition as ROI1. For the wild type sample (WT-146), an area of 92.9 µm × 89.1 µm was imaged at 10 nm/pixel using a cutting thickness (z resolution) of 40 nm. The acquired data sets were finally aligned and visualized using 3D volume rendering to highlight the RPE anomalies using Amira software (Thermo Fisher Scientific).

### 2.10. Statistical Analysis

For ERG analyses, we evaluated differences between genetically modified vs. WT control mice across flash intensities by performing repeated measures two-way ANOVA with Holm–Sidak post-hoc analysis at each time point. To evaluate functional (visual acuity, a-wave and b-wave amplitudes and implicit times) and structural (SD-OCT retinal layer thickness) parameters across time, we used a two-way repeated measures ANOVA (time X treatment conditions) with Holm–Sidak post-hoc analysis. For biochemical and quantitative immunohistochemical data, binary statistical comparisons between specific genetically modified vs. WT control group data were analyzed using an unpaired Student’s *t*-test.

## 3. Results

### 3.1. Generation of a Floxed Dhdds Mouse Line

**Figure 1:**
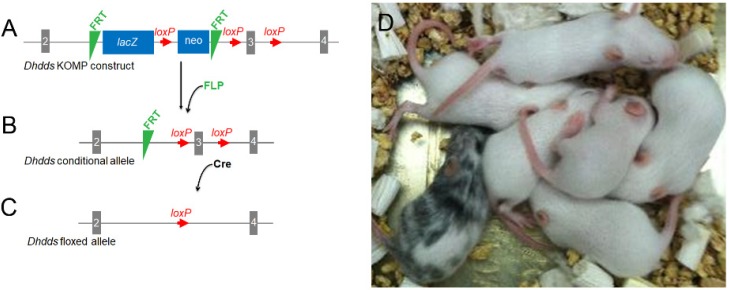
Generation of Cre-dependent *Dhdds* conditional knockout (KO) mice. (**A**) A validated *Dhdds* construct from the Knockout Mouse Project (KOMP) (U.C. Davis) was linearized and introduced into mouse ESCs. (**B**) Transformed cells were treated with FLP-FRT recombinase and PCR was used to verify lacZ cassette excision. (**C**) Clones from confirmed FRT-excised alleles were used to generate *Dhdds^flx/flx-^* mice. (**D**) Pups carrying the *Dhdds* floxed allele were identified by coat appearance.

The scheme used for the generation of a *Dhdds* conditional allele, employing a validated *Dhdds* construct (from KOMP) and mouse embryonic stem cells (ESCs), is shown in Figure 1 (see *Materials and Methods*, above). Clones from confirmed flippase recognition target (FRT)-excised alleles were used to generate *Dhdds* heterozygous and homozygous mouse lines on a C57Bl/6J background.

### 3.2. Validation of Retinal Cell Type-Specific Cre-Expressing Mouse Lines

**Figure 2:**
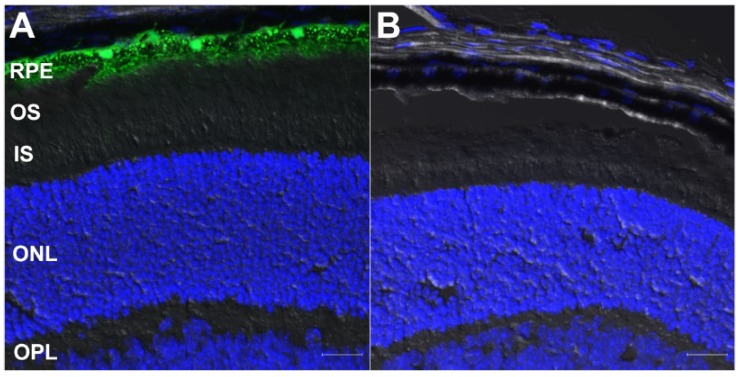
Cre recombinase-dependent ZsGreen expression (green) in mouse retina. (**A**) Retina from a postnatal (PN) one-month old CreRPE × ZsGreen reporter mouse, demonstrating ZsGreen expression specifically in retinal pigment epithelium (RPE) cells. (**B**) Retina from an age-matched, wild type (WT) mouse retina, demonstrating lack of ZsGreen expression. Nuclei counterstained with DAPI (blue). Abbreviations: IS, photoreceptor inner segment layer; OS, photoreceptor outer segment layer; ONL, outer nuclear layer; OPL, outer plexiform layer. Scale bars (both panels): 20 μm.

To assess the specificity and efficiency of the Cre recombinase in the RPE, we cross-bred transgenic mice expressing Cre recombinase (bred to homozygosity) with a ZsGreen Ai6 reporter mouse line, and then evaluated ZsGreen expression in the retina by confocal fluorescence microscopy. As expected, WT mouse retinas (a negative control) did not exhibit ZsGreen expression (Figure 2A). However, ZsGreen fluorescence was detected in Cre recombinase-positive mice specifically in the RPE layer by 1 postnatal (PN) month (Figure 2B), with >90% of the RPE cells being labeled. We subsequently confirmed that Cre recombinase continued to be expressed robustly and specifically in the RPE for >3 months (data not shown).

### 3.3. RPE-Specific Ablation of Dhdds Causes a Geographic Atrophy-Like Phenotype and Retinal Degeneration, Involving Photoreceptors

**Figure 3:**
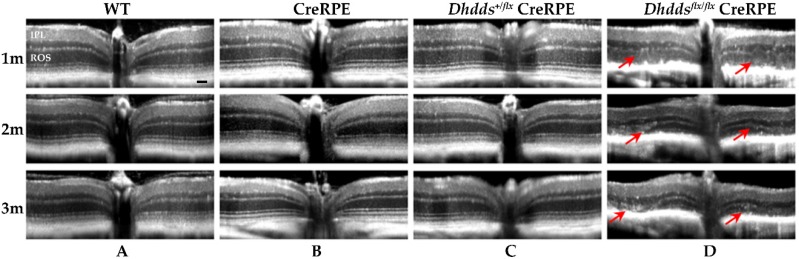
SD-OCT revealed structural changes in *Dhdds^flx/flx^* CreRPE mice at all ages. OCT scans were performed at 1, 2, and 3 months (m) postnatal for each genotype. (**A**) WT, (**B**) CreRPE, (**C**) *Dhdds*^+/flx^ CreRPE all showed normal layer stratification. (**D**) *Dhdds^flx/flx^* CreRPE showed alterations of hyper-reflectivity at all ages, indicative of pathologic changes (red arrows) and reduction in layer thickness, particularly in the outer retina. IPL, inner plexiform layer, ROS, rod outer segment. Scale bar (shown in WT 1 m panel): 50 μm, applies to all panels.

In vivo retinal imaging using SD-OCT (Figure 3) showed comparable normal layer stratification in WT, CreRPE and *Dhdds^+/flx^* CreRPE age-matched mice in each group. However, *Dhdds^flx/flx^* CreRPE mice showed altered hyper-reflectivity at all ages (indicated by red arrows, Figure 3), indicative of pathologic changes and a reduction in outer retinal layer thickness.

**Figure 4:**
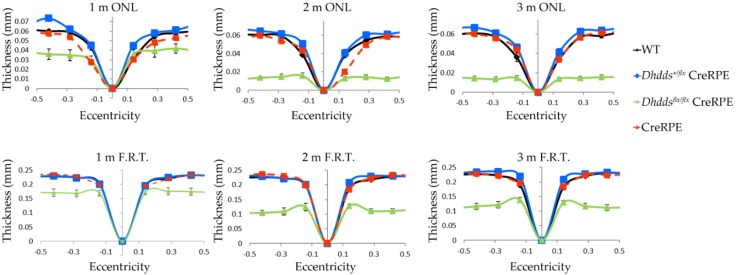
Quantitative morphometric analysis of WT, CreRPE, *Dhdds*^+/flx^ CreRPE, and *Dhdds^flx/flx^* CreRPE mice SD-OCT data. Average OCT measurements at each eccentricity revealed no significant differences when comparing WT, CreRPE and *Dhdds*^+/flx^ CreRPE mice for both outer nuclear layer (ONL) and F.R.T. thickness measurements (n = 4 for all genotypes, except for WT at one month (1 m; n = 8) and two months (2 m; n = 5). However, significant changes were observed when comparing WT and *Dhdds^flx/flx^* CreRPE mice at any age with respect to both ONL and F.R.T. values.

“Spidergram” plots of average thickness values (in mm) vs. retinal eccentricity (distance from the optic nerve head (ONH) along with vertical meridian) for the outer nuclear layer (ONL) and FRT are shown in Figure 4. No significant differences were observed when comparing WT, heterozygous, or Cre-only mice for both ONL and FRT measurements. However, *Dhdds^flx/flx^* CreRPE mice showed a significant reduction (vs. WT) in ONL and F.R.T. values at all ages analyzed (n ≥ 4, all *p* < 0.001).

**Figure 5:**
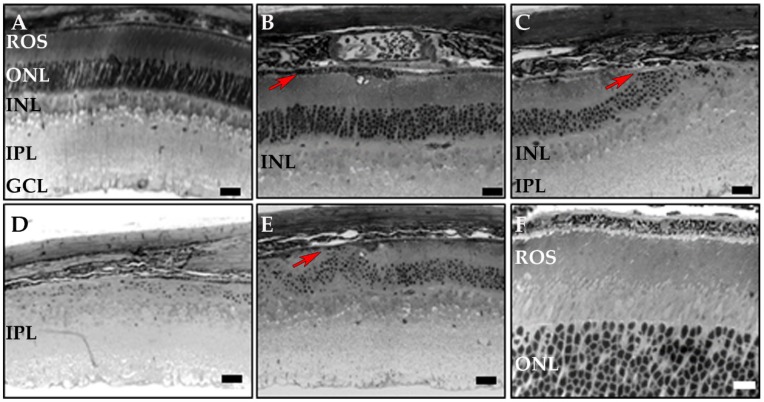
Pathology observed in *Dhdds^flx/flx^* Cre RPE mouse retina. (**A**) *Dhdds^flx/flx^* retina without Cre expression appears normal. (**B**–**F**) Five regions of a retina from a *Dhdds^flx/flx^* CreRPE mouse expressing Cre in RPE are shown. (**B**,**F**) Relatively well-preserved peripheral retina with some photoreceptor loss, outer segment (OS) shortening, and differing severity of RPE pathology are shown (arrow in panel **B** points to severely compromised RPE. (**C**,**E**) Transition zones of severe to mild retinal pathology showing loss of photoreceptors, severe compromise of RPE and external limiting membrane (ELM) descent (arrows). (**D**) A more central region of the retina showing severe cell loss in both the retina and RPE. Scale bars (all panels, except **F**): 20 μm; scale bar, panel **F**: 10 μm. ROS, rod outer segments; ONL, outer nuclear layer, INL, inner nuclear layer; IPL, inner plexiform layer, GCL, ganglion cell layer.

Histologically, at PN 3 months, light micrograph images revealed that all retina layers in mice lacking Cre expression appeared normal (Figure 5A and Supplementary Materials, Figure S1A). In contrast, the age-matched Dhdds^flx/flx^ Cre RPE mice displayed a geographic atrophy-like RPE appearance with the most degeneration observed mid-centrally throughout the retina (Supplementary Materials, Figure S1B). There were regions of well-preserved Dhdds^flx/flx^ Cre RPE retina that showed severe RPE pathology (Figure 5B). Notably, the descent of the external limiting membrane (ELM) [25] towards Bruch’s membrane was also observed (Figure 5C,E). There was a near-total loss of photoreceptors and RPE within the most affected regions (Figure 5D). Very well-preserved regions were also observed in the periphery (Figure 5F).

**Figure 6:**
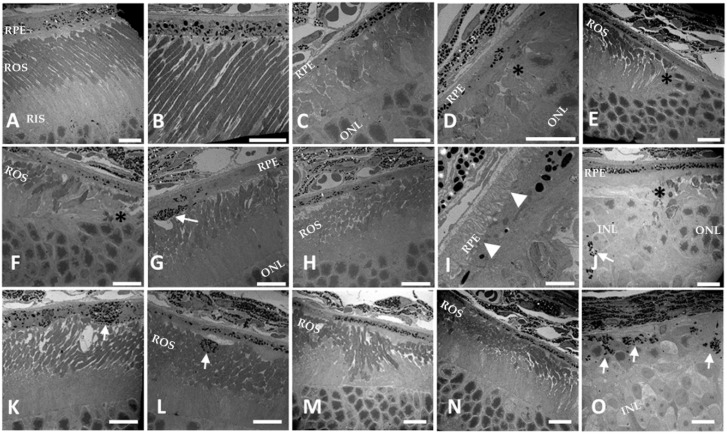
High magnification observation of *Dhdds^flx/flx^* CreRPE retina. EM images of 3m (**A**,**B**) *Dhdds^flx/flx^* and (**C**–**O**) *Dhdds^flx/flx^* CreRPE mice. Mice homozygous for the floxed *Dhdds* allele (*Dhdds^flx/flx^*) are not distinguishable from WT. (**A**) Rod outer segments are properly aligned and all retinal layers are intact (only rod inner and outer segments and the outer nuclear layer are shown). (**B**) The RPE shows normal thickness and melanin distribution. (**C**–**O**) Examples of mildly and severely compromised RPE and retina in *Dhdds^flx/flx^* CreRPE mice. Severe RPE and outer retina degeneration is concentrated mid-centrally on both sides of the optic nerve head (see histology, Figure 5, and Supplementary Materials, Figure S1). Severe RPE/PR atrophy is observed in the most affected central regions (**C**–**F**,**I**,**J**,**O**). External limiting membrane (ELM) descent (*) towards the RPE is apparent in the transition regions from severely affected to more intact regions (**D**–**F**,**J**). Transmigration of RPE cellular material into the ROS space (arrows) is also seen (**G**,**J**,**L**,**O**). Extended RPE basal fenestrations (arrowheads, **I**) are apparent in the most affected regions (**D**,**I**). Outside of the central region of severe degeneration are regions with compromised but still apparent retinal and RPE layers (**K**–**N**). Scale bars (all panels, except **I**): 10 µm; panel **I**, 2.5 µm.

Additional pathology was revealed by higher magnification EM analysis (Figure 6, panels C–O). In contrast, *Dhdds^flx/flx^* retinas without Cre expression were indistinguishable from WT retinas (Figure 6A,B). The two areas most affected showed severe RPE dystrophy with concomitant degeneration and loss of photoreceptor cells (Figure 6C,D,F,G,J,M–O). ELM descent was apparent in areas where there was a transition from milder to more severe pathology (* in panels D–F,J). RPE cell transmigration was apparent in the outer retina (arrows, panels G,J,O,L). Thus, compared to WT neural retina and RPE, the observed RPE anomalies including migration of nucleus and RPE melanosomes, displacement of the ELM, and shortened misshaped outer segments throughout the retina were seen in the *Dhdds^flx/flx^ CreRPE* mice.

To further examine the effects of *Dhdds* deletion in the RPE, serial block face-scanning electron microscopy was used to examine a ~100 μ^3^ region of the *Dhdds^flx/flx^* retina across an area of transition from milder to more severe pathology (Supplementary Materials, Videos S1–3). In WT mice, the retina appeared normal in all layers across the entire block. Examination of two regions of the *Dhdds^flx/flx^* Cre RPE retina showed areas of severe compromise as well as areas where retinal histology was more well-preserved. Transmigration of RPE nuclei and melanosomes into the photoreceptor region (subretinal space and photoreceptor outer segment layer, and even deeper, into the ONL) was also apparent.

### 3.4. Altered Scotopic and Photopic ERG Amplitudes in Dhdds^flx/flx^ CreRPE Mice

**Figure 7:**
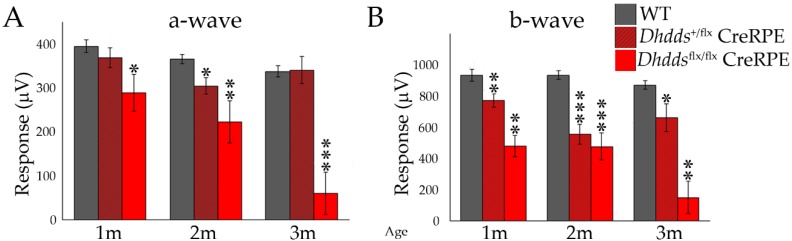
Scotopic ERG analysis of WT, *Dhdds^+/flx^* CreRPE, *Dhdds^flx/flx^* CreRPE mice. Maximum responses to saturating light stimuli showed significant decreases in (**A**) a-wave and (**B**) b-wave amplitudes for *Dhdds^+/flx^* CreRPE and *Dhdds^flx/flx^* CreRPE when compared to WT mice at 1, 2 and 3 postnatal months. Statistical significance: * *p* < 0.05, ** *p* < 0.01, *** *p* < 0.001.

Analysis by ERG (Figure 7) showed that both homozygous and heterozygous *Dhdds^flx/flx^* CreRPE mice exhibited reduced scotopic ERG a- and b-wave responses. At PN 1 month, scotopic a-wave responses (289 ± 42 µV; n = 5) were significantly reduced in *Dhdds*^flx/flx^ CreRPE mice compared to WT mice (395 ± 14 µV; n = 8; *p* < 0.05). Scotopic b-wave responses also were reduced for both *Dhdds^+/flx^* CreRPE (772 ± 42 µV; n = 20) and *Dhdds^flx/flx^* CreRPE (479 ± 69 µV; n = 5) mice, compared to WT mice (934 ± 38 µV; n = 8; *p* < 0.01). At PN 2 months of age, *Dhdds^flx/flx^* CreRPE scotopic ERG a-wave (223 ± 48 µV; n = 10) and b-wave (477 ± 88 µV; n = 10) responses and *Dhdds^+/flx^* CreRPE a-wave (305 ± 19 µV; n = 10) and b-wave (557 ± 64 µV; n = 10) responses were significantly reduced compared to the WT a-wave (366 ± 10 µV; n = 15) and b-wave (935 ± 27 µV; n = 15) responses (*p* < 0.005 for all comparisons). At PN 3 months, scotopic a-wave amplitudes were reduced only in *Dhdds*^flx/flx^ CreRPE (60 ± 48 µV; n = 6) compared to WT mice (354 ± 16 µV; n = 18; *p* < 0.001); b-wave responses were reduced in both *Dhdds^+/flx^* CreRPE (662 ± 88 µV; n = 6) and *Dhdds^flx/flx^* CreRPE (150 ± 105 µV; n = 6) mice compared to WT mice (914 ± 35 µV; n = 18) *p* < 0.05 and *p* < 0.01, respectively).

**Figure 8:**
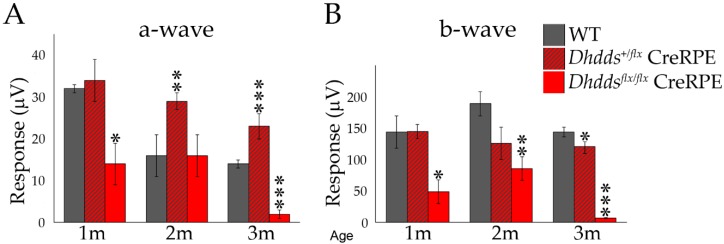
Photopic ERG analysis of WT, CreRPE *Dhdds^+/flx^*, CreRPE *Dhdds^flx/flx^* mice. (**A**) a-wave responses were significantly lower in CreRPE *Dhdds^-/-^* at 1 (n = 4) and 3 (n = 4) months postnatal compared to WT (one month (1 m), n = 5; three months (3 m), n = 9) mice. (**B**) Photopic b-wave amplitudes for *Dhdds^+/flx^* CreRPE mice showed no statistically significant differences when compared to WT mice at 1 (n = 13) and 2 (n = 4) postnatal months, but were reduced by 3 months (n = 4). In contrast, the responses of *Dhdds^flx/flx^* CreRPE mice (two months (2 m), n = 6) were significantly lower when compared to WT mice at all postnatal ages examined. Statistical significance: * *p* < 0.05, ** *p* < 0.01, *** *p* < 0.001.

The photopic a-wave and b-wave responses (Figure 8) at PN 1 month were not different when comparing WT (n = 5) and *Dhdds^+/flx^* CreRPE (n = 13) mice, however the photopic responses of *Dhdds^flx/flx^* CreRPE mice (n = 4) were significantly lower. Photopic ERG responses were significantly different only for *Dhdds^+/flx^* CreRPE a-wave (29 ± 2 µV; n = 4; *p* < 0.01), but not b-wave, responses at PN 2 months of age. In *Dhdds^flx/flx^* CreRPE mice, b-wave (86 ± 19 µV; n = 6) amplitudes were significantly reduced (*p* < 0.01), compared to WT mice (n = 13), but not the a-wave responses. At PN 3 months, *Dhdds^flx/flx^* CreRPE mice exhibited significant functional impairment in the photopic a-wave (2 ± 1 µV; n = 4) and b-wave (7 ± 1 µV; n = 4) responses compared to WT a-wave (14 ± 1 µV; n = 9) and b-wave responses (144 ± 8 µV; n = 9; *p* < 0.001 for all comparisons).

Optokinetic reflex (OKR) analysis (Supplementary Materials, Figure S3), a measure of retina-to-brain transmission (i.e., visual capacity), showed reductions in photopic (4–31%) and scotopic (8–29%) contrast sensitivity over the range of 0.031 to 0.272 c/d in PN 3-month old *Dhdds^flx/flx^* CreRPE mice (*p* < 0.05), compared to WT controls of the same age. However, no differences in spatial frequency (a measure of visual acuity) were observed between the different mouse lines. This is partly evident in the OKR scotopic and photopic plots, which show a similar high-frequency cut-off for all three mouse lines examined.

## 4. Discussion

Studies involving genetic screening of families with autosomal recessive retinitis pigmentosa have implicated a founder missense mutation (K42E) in the gene encoding DHDDS [12,26]. DHDDS is required for N-glycosylation of proteins by adding multiple copies of isopentenyl pyrophosphate (IPP) to farnesyl pyrophosphate (FPP) to produce dehydrodolichyl diphosphate (Dedol-PP), a precursor of dolichol, which is utilized as a sugar carrier in protein glycosylation in the endoplasmic reticulum [6]. Even though the generation of isoprenoid chains is complex, involving multiple enzymes and enzyme complexes [27], only DHDDS and Nogo-B receptor are required for long-chain isoprenoid synthesis (C_70-_C_120_) [28]. Previous studies have reported that mutations in the opsin gene that abolish N-linked glycosylation cause retinal degeneration [28,29,30]. To understand the basis for the ocular pathology associated with DHDDS mutation, we utilized a Cre recombinase conditional knockout (*Dhdds^flx/flx^* CreRPE) driven under the control of the RPE-specific VMD2 promoter to achieve RPE-targeted excision of the loxP-modified *Dhdds* gene, which renders the enzyme non-functional.

The VMD2 construct was originally generated to be conditional on the presence of doxycycline; however, we found that the mice displayed a phenotype in the absence of doxycycline induction. While we cannot rule out the presence of some level of doxycycline in the standard chow mouse diet, it is more likely that Cre expression was due to “leaky” expression, which bypassed doxycycline control, as confirmed in Figure 2. Since the promoters used for cell-specific targeting of Cre expression turn on after development is complete, developmental changes that would otherwise preempt retina/RPE development were not observed.

The expression of Cre recombinase in the Cre lines was confirmed using a ZsGreen reporter strategy. The *Dhdds^flx/flx^* CreRPE mice exhibited about 90% coverage of Cre expression by PN 1 month (Figure 2) which persisted up to 3 months at least. We crossed the conditional *Dhdds^flx/flx^* lines with heterozygous VMD2 Cre lines to generate homozygous *Dhdds^flx/flx^* mice with RPE-specific knockout of DHDDS expression as the model for our study.

In vivo imaging suggested that ONL thickness and F.R.T. were comparable between age-matched WT and *Dhdds^+/flx^* CreRPE mice, but significantly reduced in *Dhdds^flx/flx^* CreRPE mice. While the specific pathology is observed in much greater detail in the histological (light microscopy; Figure 5) and ultrastructural (electron microscopy; Figure 6) images provided, it is clear that the pathology indicated in the SD-OCT tomograms (Figure 3D) is consistent with the histological observations. This altered structural integrity of *Dhdds^flx/^^flx^* CreRPE retinas also corresponded to a significant decrease in a-wave and b-wave compared to age-matched WT at all flash intensities. Unexpectedly, the scotopic a- and b-waves also were reduced in *Dhdds^+/flx^* CreRPE mice, which may suggest a functional change that occurs prior to any obvious retinal structural changes and suggests that 50% DHDDS activity is insufficient in the RPE to maintain the required enzymatic activity level. Thus, carriers of *Dhdds* mutations also may develop visual defects, depending on the nature of the mutation, and other factors, such as genetic background and environment. This agrees with the rod-cone dystrophy reported in patients with autosomal recessive RP [16]. However, we cannot rule out the expression of a truncated protein that leads to a gain of function. This could explain the significant attenuation of the photoresponse in mice heterozygous for the floxed allele (Figure 7 and Figure 8). It could also possibly explain the manifestation of the disease only in ocular tissues, if the gain of function is due to specific targeting of retina-specific protein complexes. Further experimentation will be required to sort this out.

We carried out further experiments to examine the fundus of older *Dhdds^flx/flx^* CreRPE mice at 6, 8 and 10 months PN. These fundus images obtained with fluorescein angiography showed the classical signs of RP including abnormal pigmentation in 8-month old *Dhdds^flx/flx^* CreRPE mice; vascular changes, including microaneurysms, increased vessel tortuosity and attenuation, were also observed by 6 months of age (Supplementary Materials, Figure S2).

Originally it was proposed that the cause of the pathology in DHDDS-related RP patients (RP59) is defective glycosylation of rod opsin because of reduced DHDDS enzyme activity [12,26]. While this assumption may explain the classic RP symptoms observed, it fails to explain the AMD-like macular involvement. Interestingly, Lam and colleagues [11] found no N-glycosylation deficiency in RP59 patients, based upon isoelectric focusing gel analysis of plasma transferrin (a systemic glycoprotein). In addition, in a related study (Rao et al., manuscript submitted for publication), utilizing a rod photoreceptor-specific knockout of *Dhdds* in mice, we found no evidence for a resulting lack of protein glycosylation in the retina; yet, there was a rapidly progressing photoreceptor degeneration, resulting in complete loss of photoreceptors by PN 6 weeks of age. Also, in a companion article in this Special Issue [31], we generated a mouse model harboring the global K42E homozygous *Dhdds* mutation associated with RP59 patients, but observed no retinal degeneration, even out to 9 postnatal months of age. Clearly, mutations that only partially diminish enzymatic activity would be far less severe than a complete ablation of the gene encoding the enzyme.

## 5. Conclusions

From these observations, we conclude that targeted ablation of *Dhdds* selectively in RPE cells results in perturbation of DHDDS-dependent processes, resulting in structural and functional deficits in both the RPE and photoreceptors, in a manner resembling geographic atrophy. The degeneration progresses relatively slowly (compared to the rod-specific *Dhdds* ablation model) over the course of a few months, rather than weeks.

## Supplementary Materials

The following are available online at https://www.mdpi.com/2073-4409/9/3/771/s1, Figure S1: Stitched images of *Dhdds^flx/flx^* and *Dhdds^flx/flx^* Cre RPE mouse retina, Figure S2: Fundus imaging and fluorescein angiography (FA), Figure S3: Contrast sensitivity and spatial frequency assessment in WT, *Dhdds^flx/flx^*, and *Dhdds^flx/flx^* Cre RPE mice, Video S1: Serial Block-Face Scanning Electron Microscopy of WT *Dhdds^flx/flx^*, Video S2: Serial Block-Face Scanning Electron Microscopy of region 1 of *Dhdds^flx/flx^* Cre RPE, Video S3: Serial Block-Face Scanning Electron Microscopy of region 2 of *Dhdds^flx/flx^* Cre RPE.
