# Supplementary material for: Selective Ablation of Dehydrodolichyl Diphosphate Synthase in Murine Retinal Pigment Epithelium (RPE) Causes RPE Atrophy and Retinal Degeneration
Source: Cells. 2020 Mar 21;9(3):771. doi: 10.3390/cells9030771 (PMC7140717; doi:10.3390/cells9030771)
Supplement: Supplementary file 1 [file cells-09-00771-s001.pdf]

Supplementary Materials for

Selective ablation of dehydrodolichyl diphosphate synthase in murine retinal pigment epithelium (RPE) causes RPE atrophy and retinal degeneration.

Marci L. DeRamus, Stephanie Davis, Sriganesh Ramachandra Rao, Cyril Nyankerh, Delores Stacks, Timothy W. Kraft, Steven J. Fliesler, and Steven J. Pittler

A

B

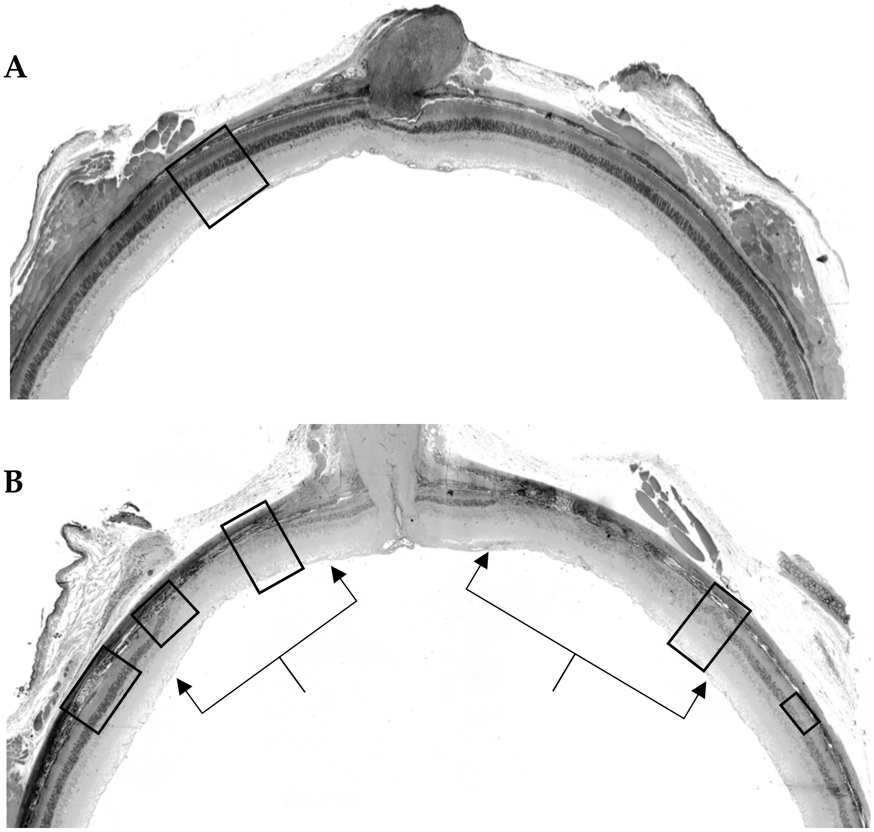

Figure S1. Stitched images of *Dhdds*$^{flx/flx}$ and *Dhdds*$^{flx/flx}$ CreRPE mouse retina. A) *Dhdds*$^{flx/flx}$ 3 mos. mouse retina. The presence of loxP sites flanking exon 2 does not affect the structure of the retina, which appears indistinguishable from WT. B) *Dhdds*$^{flx/flx}$ CreRPE 3 mos. mouse retina. Regions of central retina on both sides of the optic nerve (delimited by arrows) are most affected. Boxes in A) and B) denote regions of interest shown in Fig. 5.

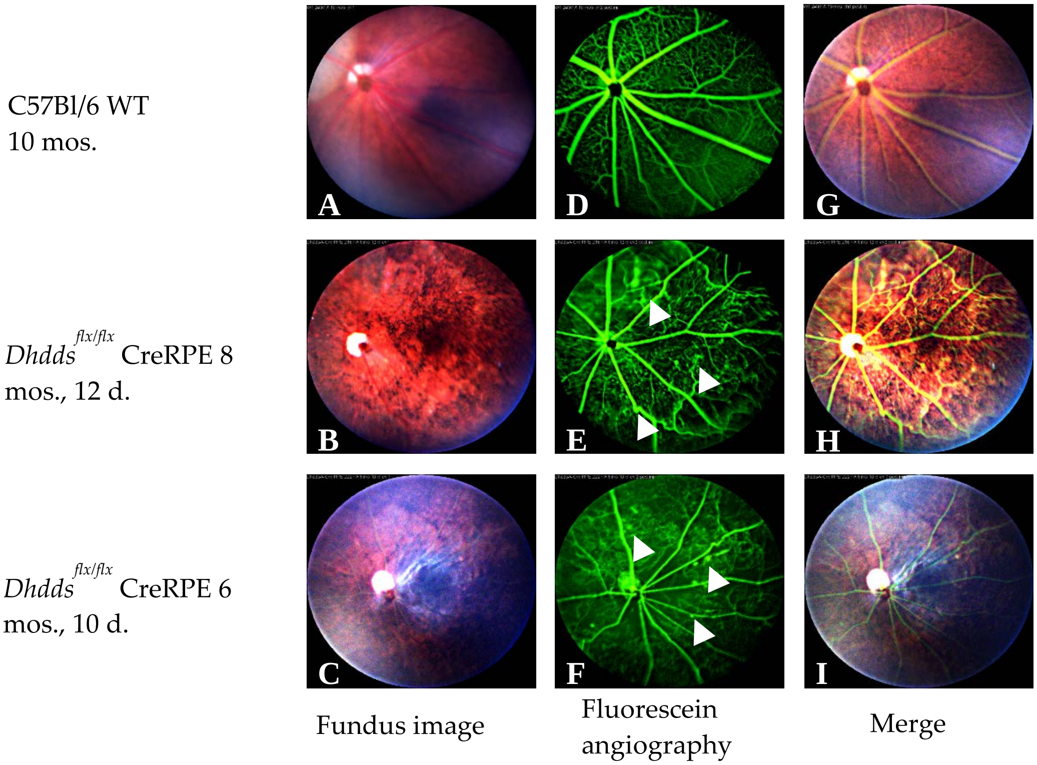

Figure S2. Fundus imaging and fluorescein angiography (FA) (**A-C**) Fundus imaging. Compared to WT (**A**), the 6- and 8-mo old *Dhdds*$^{flx/flx}$ CreRPE mice appear to have a patchy distribution of pigment. FA (**D-F**). The WT image (**D**) shows an intact vascular network in contrast to apparent aneurisms and micro-aneurisms, vessel tortuosity, and possible neovascularization in the Cre expressing mice (**E, F**). Arrowheads in (**E**) and (**F**) point to a few areas of the vasculature that are affected among numerous changes that are apparent. (**G-I**) Fundus and FA images merge. Merging of the FA and fundus images indicates no correlation of pigmentary and vasculature changes.

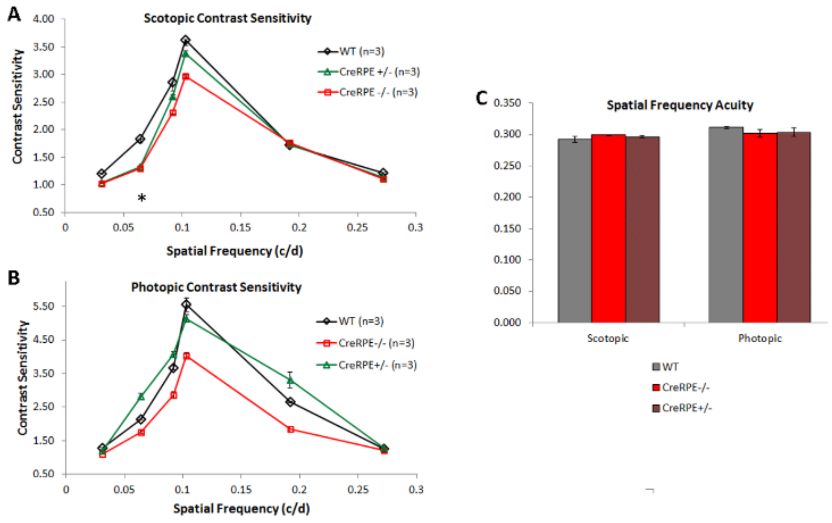

Figure S3. Contrast sensitivity and spatial frequency assessment in WT, *Dhdds*$^{flx/flx}$, and *Dhdds*$^{flx/flx}$ CreRPE mice. Contrast sensitivity responses in scotopic (A) and (B) photopic conditions shows a reduction in contrast sensitivity in 3-month-old *Dhdds*$^{flx/flx}$ CreRPE compared to WT. C) No significant differences were observed for spatial acuity measures among the three groups.

Video S1: Serial Block Face- Scanning Electron Microscopy of WT *Dhdds*$^{flx/flx}$.

Video S2: Serial Block Face- Scanning Electron Microscopy of region 1 of D*hdds*$^{flx/flx}$ CreRPE.

Video S3: Serial Block Face- Scanning Electron Microscopy of region 2 of D*hdds*$^{flx/flx}$ CreRPE.

Videos description:

The videos show the serial images obtained for two regions of the *Dhdds*$^{flx/flx}$ CreRPE and one region of WT mouse retinas. Each video begins with a run through of each grey scale image and each video ends with a pseudo-colored view of the image combined to generate a 3D volume. Colors were randomly assigned to different ranges of pixel intensities using volume rendering in the Amira software. The end of the videos filters the image to show only the areas of greatest pixel intensity range.
